# ASK1 Mediates Nur77 Expression in T-Cell Receptor Mediated Thymocyte Apoptosis

**DOI:** 10.3390/cells9030585

**Published:** 2020-03-01

**Authors:** Jianxin Huo, Shengli Xu, Kong-Peng Lam

**Affiliations:** 1 Bioprocessing Technology Institute, Agency for Science, Technology and Research, Singapore 138668, Singapore; huo_jianxin@bti.a-star.edu.sg (J.H.); xu_shengli@bti.a-star.edu.sg (S.X.); 2 Department of Physiology, Yong Loo Lin School of Medicine, National University of Singapore, Singapore 117593, Singapore; 3 Department of Microbiology and Immunology, Yong Loo Lin School of Medicine, National University of Singapore, Singapore 117545, Singapore; 4 School of Biological Sciences, Nanyang Technological University, 60 Nanyang Drive, Singapore 637551, Singapore

**Keywords:** ASK1, p38, JNK, Nur77, caspase, Akt, thymocyte, TCR mediated apoptosis

## Abstract

Apoptosis signal-regulating kinase 1 (ASK1) is a mitogen-activated protein kinase kinase kinase (MAPKKK) that activates downstream JNK and p38 mitogen-activated protein kinase (MAPK) to relay death signals into cells in response to various environmental stress. However, whether ASK1 plays a role in T cell receptor (TCR)-mediated apoptosis of thymocytes is unclear. Here, we show that ASK1 is activated upon TCR stimulation and plays an important role in TCR-mediated apoptosis of thymocytes by triggering downstream JNK and p38 signaling cascades. Mechanistically, ASK1-JNK/p38 signaling leads to the upregulation of neuron-derived clone 77 (Nur77), a critical pro-apoptotic protein involved in TCR-mediated apoptosis of thymocytes. Furthermore, we demonstrate that the activation of ASK1 is negatively modulated by Akt upon TCR stimulation. Thus, our results identify a previously unappreciated signaling mechanism involving ASK1 in TCR-mediated apoptosis of thymocytes.

## 1. Introduction

The T cell receptor (TCR) on thymocytes balances sensitivity and specificity in recognizing self-peptide and major histocompatibility complex (self-pMHC) during the process of positive and negative selection in developing T cells [1,2]. Thymocytes with TCR of low- or moderate- affinity for self-pMHC receive survival signals and are positively selected. On the other hand, thymocytes expressing high-affinity TCRs for self-pMHC are eliminated through TCR-mediated apoptosis. This process is crucial in establishing the central tolerance of T cells [3,4]. Complex intracellular signaling pathways are involved in this process to convey signals from the TCR to trigger the cell death machinery that results in the apoptosis of thymocytes [5,6]. Amongst the multiple signaling cascades, c-Jun NH2-terminal kinase (JNK) and p38 MAPK pathways have been shown or implicated as being involved in TCR-mediated apoptosis [7]. JNK and p38 are two members of the mitogen-activated protein kinase (MAPKs) family and are known to be preferentially activated by cytotoxic stress signals, including UV irradiation, reactive oxygen species, and inflammatory cytokines. JNK has three isoforms, with JNK1 and JNK2 ubiquitously expressed and JNK3 restricted to the brain [8]. It has been shown that JNK2 is required for the TCR-mediated apoptosis of thymocytes [9]. The ablation of JNK1 also causes thymocytes to be partially resistant to TCR-mediated apoptosis in vivo [10]. Although p38 MAPK has not been shown directly to be required for TCR-mediated apoptosis of thymocytes due to embryonic lethality of p38 MAPK mutant mice, studies using mice deficient in MAPK kinase 6 (MKK6), which is the upstream kinase predominantly regulating p38 activation, suggested that MKK6-p38 pathway is involved in TCR-mediated apoptosis of thymocytes [11]. 

JNK and p38 MAPK are activated through a sequential cascade composing three classes of serine/threonine kinases, MAPK kinase kinase (MAPKKK or MAP3K), MAPK kinase (MAPKK or MKK), and MAPK. Upon activation, MAP3K becomes phosphorylated and in turn phosphorylates and activates MAPKK, which further activates MAP kinase. A number of MAP3Ks, including TAK1, MTK1, MLK2/MST, MLK3/PTK/SPRK, and DLK/MUK/ZPK, have been shown to activate JNK/p38 MAPKs through various MAPKKs [12]. For instance, TAK1 can phosphorylate and activate MKK6 and MKK7, leading to p38 and JNK activation [13]. The deletion of TAK1 sensitizes thymocytes to apoptosis and leads to impaired T cell development [14]. Apoptosis signal-regulating kinase 1 (ASK1, or MAPKKK5), which is ubiquitously expressed, is another important member of the MAP3Ks and plays critical roles in many physiological processes. ASK1 is known to activate JNK and p38 MAPKs through activating MKK4/MKK7-JNK and MKK3/MKK6-p38 signaling cascades, respectively, in response to stress signals [15,16]. Particularly, activated ASK1 binds MKK4/7 directly and activates these MKKs to initiate JNK/p38 MAPK signal transduction [17].

Studies have also demonstrated that the ASK1-JNK/p38 cascades are important for cell death induced by various cytotoxic stress signals [18] and that PI3K/Akt negatively regulates ASK1 activation through phosphorylation of the serine 83 residue of ASK1 [19]. It has been demonstrated that ASK1-deficient thymocytes exhibited decreased Fas-induced apoptosis and defective activation of JNK and p38 MAPKs [20]. However, it is not clear whether ASK1 is required for JNK/p38 MAPK activation upon TCR engagement and, if yes, whether ASK1-JNK/p38 signaling axes are involved in TCR-induced apoptosis of thymocytes, and how ASK1 activation is regulated. 

Another important molecule implicated in thymocyte apoptosis is the orphan nuclear receptor Nur77, which was proapoptotic and induced by TCR engagement [21,22]. Our previous study demonstrated that Akt inhibition led to increased Nur77 expression in TCR-stimulated thymocyte apoptosis [23]. More recently, a study showed that JNK activation is required for the nuclear export of Nur77 and that p38 MAPK is essential for mitochondrial targeting of Nur77 to initiate the Nur77-Bcl-2 apoptotic pathway in cancer cells [24]. However, it is not clear if TCR-mediated Nur77 upregulation is regulated by ASK1-JNK/p38 signaling in thymocytes.

Here, we demonstrate that ASK1 is activated in TCR-stimulated thymocytes and is required for their apoptosis through activating JNK/p38 MAPKs. Interestingly, our data further revealed that ASK1-JNK/p38 cascades upregulate Nur77 protein expression and result in the activation of caspases and apoptosis of thymocytes. We also discovered that Akt functions upstream to negatively modulate the activation of ASK1 upon TCR engagement. Thus, our study reveals a previously unappreciated Akt-ASK1-JNK/p38-Nur77 signaling pathway during TCR-mediated apoptosis of thymocytes. 

## 2. Materials and Methods

### 2.1. Mouse

Wild-type C57BL/6, *jnk1^−/−^* (from Jackson Laboratories, Bar Harbor, ME, USA) and *faim*^−/−^ mice [25] were housed under a 12 h light–dark cycle and given normal chow (4% of crude fat, 11% calories from fat, #1320 mod., Altromin). Mice aged 8 to 16 weeks old of both sexes were used, and all animal procedures were approved (IACUC 171281) by the Institutional Animal Care and Use Committee of Agency for Science, Technology and Research, Singapore.

### 2.2. Antibodies and Reagents 

The following antibodies were used for immunoblotting: rabbit anti-JNK1/2 (D-9) and anti-p38(N-20) were purchased from Santa Cruz Biotechnology (Santa Cruz, CA, USA); rabbit anti-cleaved Caspase-3 (Asp175), rabbit anti-phospho-ASK1 (T845), anti-ASK1, anti-phospho-p38 and anti-phospho-JNK, anti-phospho-MKK3 (Ser189)/MKK6 (Ser207) (22A8), anti-phospho-SEK1/MKK4 (ser257) (C36C11), anti-MKK3, and anti-SEK1/MKK4 were purchased from Cell Signaling Technology (Danvers, MA, USA), and anti-Nur77 was purchased from BD Biosciences (San Jose, CA, USA). The rabbit anti-FAIM polyclonal antibody was raised in-house against the full length protein of mouse FAIM [23]. ASK1-specific siRNA was purchased from Santa Cruz Biotechnology. ASK1 inhibitor 2,7-Dihydro-2,7-dioxo-3H-naphtho[1,2,3-de]quinoline-1-carboxylic acid ethyl ester was purchased from Axon Medchem LLC, Reston, VA, USA. Akt inhibitor 1,3-dihydro-1-(1-((4-(6-phenyl-1H-imidazo[4,5-g]quinoxalin-7-yl)phenyl)methyl)-4-piperidinyl)-2H-benzimidazol-2-one trifluoroacetate salt, JNK inhibitor 1,9-Pyrazoloanthrone (SP600125), and p38 inhibitor 4-(4-Fluorophenyl)-2-(4-methylsulfinylphenyl)-5-(4-pyridyl)-1H-imidazole (SB203580)1, 9-Pyrazoloanthrone (SP600125) were purchased from Sigma-Aldrich, St. Louis, MO, USA.

### 2.3. Cell Viability Analysis

Single-cell suspensions were prepared from thymus of mice as previously described [23]. The viability of thymocytes was examined using Annexin V apoptosis detection kit (BD Pharmingen, San Diego, CA, USA), following the manufacturer’s instruction. Briefly, 0.1 × 10^6^/mL cells were harvested, washed once with cold phosphate-buffered saline, and stained with Annexin V–fluorescein isothiocyanate and propidium iodide (PI) in 1× Annexin staining buffer followed by a flow cytometric analysis. Live cells were determined as Annexin V/PI double negative cells. Data were collected on a LSR II flow cytometer (BD Pharmingen) and analyzed with Flowjo (Treestar). All experiments were performed in triplicate.

### 2.4. Immunoblotting

The preparation of samples and immunoblotting were performed as previously described [26]. Briefly, whole cell lysates were prepared using lysis buffer (10 mM Tris-HCl, pH 8.0, 150 mM NaCl, 1mM EDTA, 1% Igepal CA-630, 0.2 mM Na_3_VO_4_, and a Protease Inhibitor Cocktail, Roche, Basel, Switzerland). The protein concentration was measured by a colorimetric assay (Bio-Rad, Hemel Hempstead, Hertfordshire, UK), and equal amounts of proteins were loaded onto SDS-PAGE. After a transfer to polyvinylidene difluoride (PVDF) membranes, the proteins were probed with specific monoclonal or polyclonal primary antibodies (1 μg/mL), followed by horseradish peroxidase-conjugated secondary antibodies. The membranes were washed and visualized with a SuperSignal West Pico/Dura chemiluminescent substrate (Pierce, Rockford, IL, USA).

### 2.5. Statistical Analysis

All experiments were performed at least three times. Data are presented as the mean ± SEM. A statistical comparison of the data was performed using Student’s *t* test. A group difference with *p* < 0.05 was considered statistically significant.

## 3. Results

### 3.1. ASK1 Is Activated and Required for Thymocyte Apoptosis upon TCR Engagement

**Figure 1:**
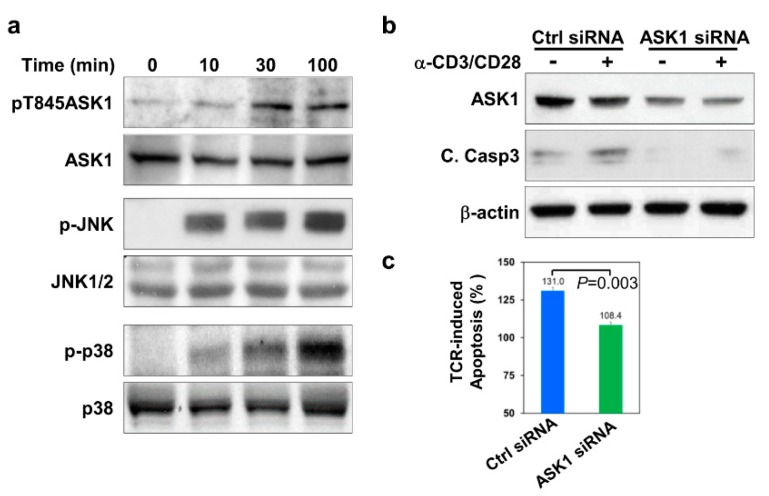
ASK1 is activated and involved in the apoptosis of thymocytes upon TCR stimulation. (**a**) Activation of ASK1 and JNK/p38 in thymocytes upon TCR-engagement. Thymocytes were stimulated with anti-CD3 (10 μg/mL) and anti-CD28 (1 μg/mL) antibodies for various periods of time, as indicated. Whole cell lysates were prepared and subjected to immunoblotting to examine the phosphorylation status of ASK1 at Thr845, JNK at Thr183/Tyr185, and p38 at Thr180/Tyr182, respectively. The amount of ASK1, JNK1/2, and p38 was also detected and included as loading controls for the individual protein. (**b**) Knockdown of ASK1 and its effect on TCR-induced caspase-3 activation in thymocytes. Thymocytes were transfected with control siRNA (ctrl siRNA) or ASK1-specific siRNA (ASK1 siRNA), followed by culture without or with anti-CD3 (10 μg/mL) and anti-CD28 (1 μg/mL) antibodies for 16 h. Whole cell lysates were prepared and subjected to immunoblotting for the detection of ASK1 and the activated or cleaved form of caspase-3. An anti-β-actin blot was included as the loading control. (**c**) Suppression of TCR-induced apoptosis of thymocytes by ASK1 knockdown. Thymocytes were transfected with ctrl siRNA or ASK1 siRNA and treated with anti-CD3 (10 μg/mL) and anti-CD28 (1 μg/mL) antibodies for 16 h. Cells were harvested and stained with Annexin V and propidium iodide (PI), followed by a flow cytometric analysis. The percentage of TCR-induced apoptosis of thymocytes was expressed as: (percentage of dead cells with TCR treatment - percentage of dead cells without TCR treatment)/percentage of dead cells without TCR treatment. The data shown are representative of more than three independent experiments (**a**,**b**). The bar graph is shown as the mean ± SD (*n* = 3).

As a ubiquitously expressed MAP3K, ASK1 is essential for stress-induced apoptosis by relaying upstream signals via MKK4/MKK7-JNK and MKK3/MKK6-p38 MAPK pathways to activate the apoptotic machineries in various cell types under different stress conditions [15]. Given that JNK and p38 MAPK are activated by TCR engagement and are important for TCR-mediated apoptosis of thymocytes [27], it is intriguing to know if ASK1 is also activated by TCR stimulation and therefore contributes to the TCR-mediated apoptosis of thymocytes. We first stimulated thymocytes from wild-type mice with plate-bound anti-CD3 and anti-CD28 antibodies, which mimic TCR stimulation, and examined the phosphorylation status of ASK1. Upon stimulation, ASK1 is phosphorylated at the threonine 845 (T845) residue, which is important for its activation [20]. When TCRs on thymocytes were stimulated with anti-CD3 and anti-CD28 antibodies, it was found that ASK1 became phosphorylated at the T845 residue at 10 min post-stimulation and that the phosphorylation was sustained for up to 100 min (Figure 1a). Interestingly, the activation of JNK and p38 MAPKs, as indicated by their phosphorylation at the T183/Y185 and T180/Y182 residues on JNK and p38 respectively, also exhibited a similar kinetics to that of ASK1 and peaked at the 100 min time-point in TCR-stimulated thymocytes (Figure 1a). These results suggest that ASK1 is activated together with JNK and p38 upon TCR stimulation in thymocytes.

Next, we determined if ASK1 is involved in TCR-mediated apoptosis of thymocytes. We first knocked down ASK1 in thymocytes using ASK1-specific siRNA and subsequently stimulated them with anti-CD3 and anti-CD28 antibodies. ASK1-specific siRNA could reduce the protein level of ASK1 by ~50%, as compared to scrambled control siRNA (Figure 1b, lanes 1, 2 versus lanes 3, 4, and Supplementary Figure S1). TCR-stimulation led to a 30% increase in the apoptosis of scrambled siRNA-transfected thymocytes after 24 h of culture compared with untreated cells (Figure 1c). In contrast, when thymocytes with knocked-down ASK1 were treated with anti-CD3 and anti-CD28 antibodies, they displayed only minimally increased apoptosis (~8%) compared to that of untreated thymocytes (Figure 1c). Consistent with suppressed TCR-induced apoptosis, the activation of caspase-3, as indicated by the amount of its cleaved form, was also largely undetected in TCR-stimulated thymocytes with ASK1 expression was knocked down (Figure 1b and Supplementary Figure S1). Taken together, these data suggest that ASK1 is activated by TCR stimulation and is required for TCR-mediated caspase-3 activation and the apoptosis of thymocytes.

### 3.2. ASK1- JNK/p38 Pathways Are Important for TCR-Induced Apoptosis of Thymocytes

**Figure 2:**
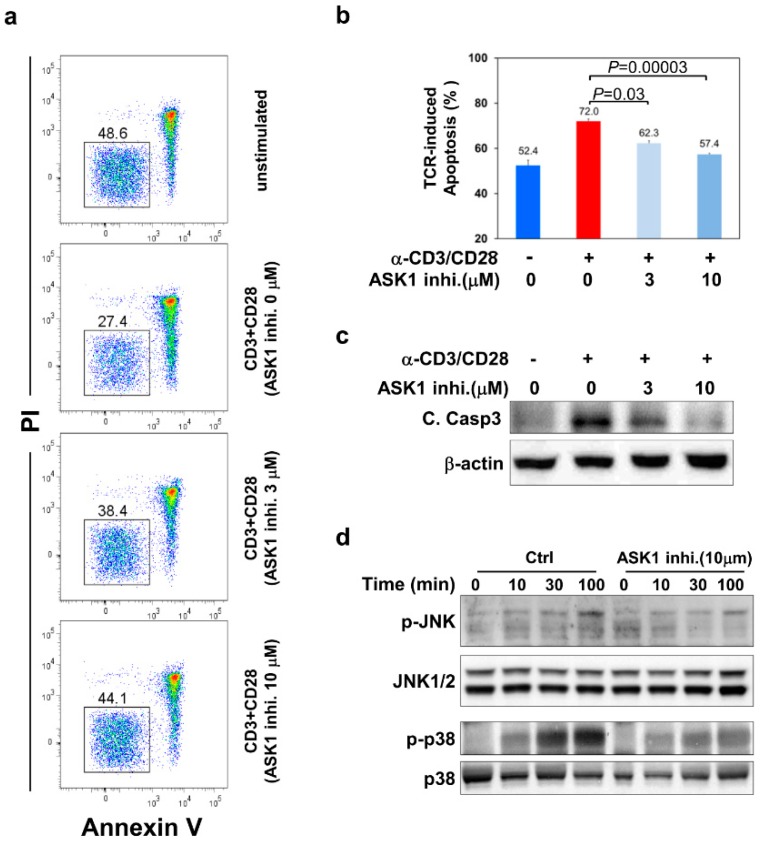
The ASK1- JNK/p38 pathways are critical for TCR-induced apoptosis of thymocytes. Thymocytes were either unstimulated or stimulated with anti-CD3 (10 μg/mL) and anti-CD28 (1 μg/mL) antibodies for 16 h in the absence or presence of 3 or 10 μM of the ASK1 inhibitor (2,7-Dihydro-2,7-dioxo-3H-naphtho[1,2,3-de]quinoline-1-carboxylic acid ethyl ester, ASK1 inhi.). (**a**) Cells were harvested and stained with Annexin V and PI, followed by flow cytometric analysis to detect Annexin V^-^PI^-^ live cells. (**b**) The percentage of dead cells (100% - live cells %) was quantified based on the flow cytometric analysis. (**c**) Attenuation of TCR-induced caspase-3 activation by the inhibition of ASK1. Whole cell lysates from thymocytes cultured under the same conditions as in (**a**) were extracted and subjected to immunoblotting to determine caspase-3 activation by detecting the amount of cleaved caspase-3 (C.Casp3). An anti-β-actin blot was included as the loading control. (**d**) The TCR-induced activation of JNK and p38 is suppressed by the inhibition of ASK1. Thymocytes were stimulated with anti-CD3 (10 μg/mL) and anti-CD28 (1 μg/mL) antibodies for different periods of time, as indicated in the absence or presence of 10 μM of the ASK1 inhibitor. Whole cell lysates were harvested and subjected to immunoblotting to examine the phosphorylation status of JNK and p38. The amount of JNK and p38 was also detected and included as loading controls. The data shown are representative of more than three independent experiments (**a**,**c**,**d**). The bar graph is shown as the mean ± SD (*n* = 3 for **b**).

As ASK-1 is the upstream MAP3K for JNK/p38 MAPKs in various cytotoxic stress-induced cell death pathways, it is intriguing to know if ASK1 also acts upstream of JNK/p38 MAPK in the TCR-induced apoptosis of thymocytes, especially as it was noted that they had similar activation kinetics upon TCR stimulation (Figure 1a). To this end, we treated thymocytes with an ASK1-specific inhibitor 2,7-Dihydro-2,7-dioxo-3H-naphtho[1,2,3-de] quinoline-1-carboxylic acid ethyl ester to inhibit its kinase activity, followed by stimulation with anti-CD3 and anti-CD28 antibodies to induce thymocyte apoptosis and the examination of JNK/p38 activation. After TCR stimulation, thymocytes pretreated with 3 μM of the ASK1 inhibitor exhibited significantly improved survival compared to those without pretreatment with ASK-1 inhibitor (cell viability: 38.4% vs. 27.4%; apoptosis: 62.3% vs. 72.0% in Figure 2b) (Figure 2a). The inhibitory effect of the ASK1 inhibitor on TCR-induced apoptosis was more prominent at 10 μM concentration (cell viability: 44.1% vs. 27.4% in Figure 2a; apoptosis: 57.4% vs. 72.0% in Figure 2b), but it became less significant at 30 μM, which could be due to its toxicity side effect (data not shown). Consistently, the TCR-induced activation of caspase-3 was also reduced in thymocytes when they were pretreated with 3 or 10 μM of the ASK1 inhibitor (Figure 2c).

Next, we examined the activation status of JNK and p38 MAPKs at different time-points upon TCR stimulation in thymocytes with or without pretreatment with the ASK1 inhibitor. Without ASK1 inhibition, JNK and p38 became activated upon TCR engagement, as indicated by their phosphorylation, which peaked at 100 min after stimulation (Figure 2d). However, in thymocytes pretreated with 10 μM of the ASK-1 inhibitor, TCR-induced activation of JNK and p38 was significantly reduced at all examined time-points (Figure 2d and Supplementary Figure S2). These results suggest that ASK1 acts upstream of JNK/p38 signaling pathways during TCR-induced apoptosis of thymocytes.

### 3.3. ASK1-JNK/p38 Signaling Axes Regulate the Level of Nur77 in TCR-Stimulated Thymocytes

Although the data so far suggested that ASK1-JNK/p38 signaling pathways play an important role in TCR-induced apoptosis of thymocytes, it was still unclear how these signaling axes eventually triggered caspase activation and led to the death of thymocytes. It is known that ASK1-induced apoptosis is executed through targeting the mitochondria-dependent apoptotic pathway [28]. Amongst the molecules known to be important for TCR-induced apoptosis and the negative selection of thymocytes is neuron-derived clone 77 (Nur77), which was shown to target the mitochondria-dependent apoptotic pathway by associating with Bcl-2 and converting Bcl-2 into a proapoptotic molecule to initiate caspase activation [29]. Previously, ERK MAPKs have been shown to regulate Nur77 in the activation-induced cell death of T cells or retinoid-induced apoptosis of cancer cells [6,30,31,32,33]. Thus, we asked if Nur77 was involved in ASK1/JNK/p38-mediated apoptosis of thymocytes upon TCR engagement.

**Figure 3:**
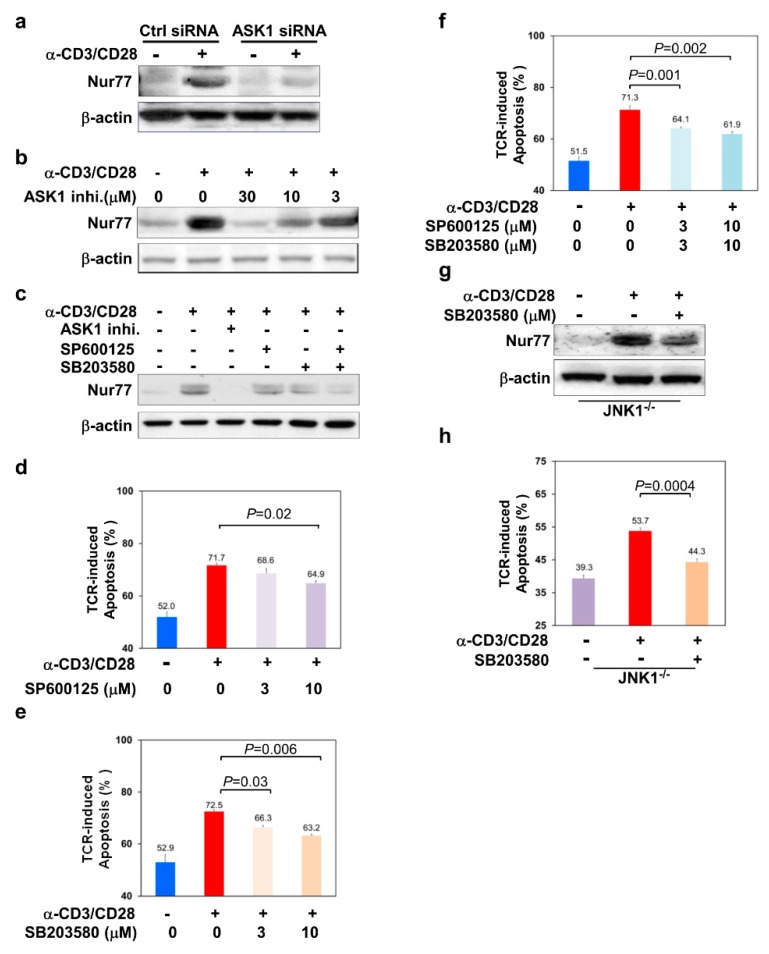
The ASK1-JNK/p38 signaling axes are important for TCR-induced Nur77 upregulation and thymocyte apoptosis. (**a**,**b**) ASK1 is required for TCR-induced upregulation of Nur77 in thymocytes. Thymocytes were (**a**) transfected with ctrl siRNA or ASK1 siRNA or (**b**) treated with various concentrations of ASK1 inhibitor as indicated, followed by culture without or with anti-CD3 (10 μg/mL) and anti-CD28 (1 μg/mL) antibodies for 16 h. Whole cell lysates were prepared and subjected to immunoblotting to detect Nur77. Anti-β-actin blots were included as loading controls. (**c**) Suppression of TCR-induced Nur77 upregulation by the inhibition of ASK1-JNK/p38 signaling cascades in thymocytes. Thymocytes were cultured without or with anti-CD3 (10 μg/mL) and anti-CD28 (1 μg/mL) antibodies in the absence or presence of 10 μM of the ASK1 inhibitor, 10 μM of the JNK inhibitor (SP600125), or 10 μM of the p38 inhibitor (SB203580) for 16 h. Whole cell lysates were prepared and subjected to immunoblotting to detect Nur77. An anti-β-actin blot was included as the loading control. (**d** –**f**) Suppression of TCR-induced apoptosis of thymocytes by inhibition of JNK/p38 MAPK signaling cascades. Thymocytes were cultured without or with anti-CD3 (10 μg/mL) and anti-CD28 (1 μg/mL) antibodies in the absence or presence of 3 or 10 μM of (**d**) JNK inhibitor SP600125, (**e**) p38 inhibitor SB203580, or (**f**) both inhibitors for 16 h. Cells were harvested, and the level of thymocyte apoptosis was analyzed as described in Figure 2a. (**g**, **h**) Suppression of (**g**) TCR-induced Nur77 upregulation and (**h**) apoptosis of JNK1-defective thymocytes by p38 inhibition. JNK1-defective thymocytes were cultured either without or with anti-CD3 (10 μg/mL) and anti-CD28 (1 μg/mL) antibodies in the absence or presence of the p38 inhibitor SB203580 (10 μM) for 16 h. Whole cell lysates were prepared and subjected to immunoblotting for the detection of Nur77 levels. An anti-β-actin blot was included as the loading control. The apoptosis of thymocytes was analyzed by the method described in Figure 2a. The data shown are representative of more than three independent experiments (**a**–**c**,**g**). The bar graph is shown as mean ± SD (*n* = 3 for **d**–**f**,**h**).

We first knocked down ASK1 in thymocytes and examined the protein level of Nur77 after stimulation with anti-CD3 and anti-CD28 antibodies. In control scramble siRNA-transfected thymocytes, TCR stimulation resulted in a significant increase in Nur77 protein level (Figure 3a, lane 2). In contrast, the upregulation of Nur77 was significantly suppressed in thymocytes transfected with ASK1-specific siRNA (Figure 3a, lane 4). Similarly, the upregulation of Nur77 was also dampened when thymocytes were pretreated with different concentrations of the ASK1 inhibitor (Figure 3b). These results suggest that ASK1 is required for TCR-mediated Nur77 upregulation in thymocytes.

Next, we asked if JNK/p38 MAPK pathways were required for TCR-mediated Nur77 upregulation downstream of ASK1. We used the JNK- and p38-specific inhibitors, 1, 9-Pyrazoloanthrone (SP600125) and 4-(4-Fluorophenyl)-2-(4-methylsulfinylphenyl)-5-(4-pyridyl)-1H-imidazole (SB203580) to inhibit these two MAPKs respectively and examined their effect on TCR-mediated Nur77 upregulation. Without treatment with any chemical inhibitor, Nur77 was significantly upregulated in thymocytes upon TCR stimulation (Figure 3c, lane 2, Supplementary Figure S2). In contrast, TCR-mediated upregulation of Nur77 was partially dampened by treatment of either the JNK- or p38-specific inhibitor (Figure 3c, lanes 4 and 5, Supplementary Figure S2). Of note, the co-inhibition of JNK and p38 pathways had a stronger inhibitory effect on Nur77 upregulation as compared to the inhibition of a single pathway (Figure 3c, lane 6, Supplementary Figure S2), suggesting that both ASK1-JNK and ASK1-p38 pathways contribute to Nur77 upregulation.

Nur77 is known to play an important role in TCR-mediated apoptosis of thymocytes [32,33]. Consistent with the partial inhibition of Nur77 upregulation, the treatment of thymocytes with the JNK inhibitor SP600125 improved the cell viability of TCR-stimulated thymocytes by 19.4% (35.1% vs. 28.3%) compared to that without JNK inhibition (Figure 3d). Similarly, p38 MAPK inhibitor SB203580 also significantly enhanced the viability of TCR-stimulated thymocytes by 18.4% (33.7% vs. 27.5%) and 25.3% (36.8% vs. 27.5%) at 3 μM and 10 μM concentrations, respectively (Figure 3e). Interestingly, TCR-induced apoptosis of thymocytes was further suppressed when both JNK and p38 were combinatorially inhibited (Figure 3f). Furthermore, when we inhibited p38 in JNK1-deficient thymocytes using the p38-specific inhibitor SB203580, both TCR-induced upregulation of Nur77 and apoptosis were significantly reduced in JNK1-deficient thymocytes (Figure 3g,h). Taken together, these results suggest that both JNK and p38 MAPKs act downstream of ASK1 to regulate the level of Nur77 and TCR-induced apoptosis of thymocytes.

### 3.4. Akt Negatively Regulates ASK1-MKK4-JNK/p38-Nur77 Signaling Pathway

**Figure 4:**
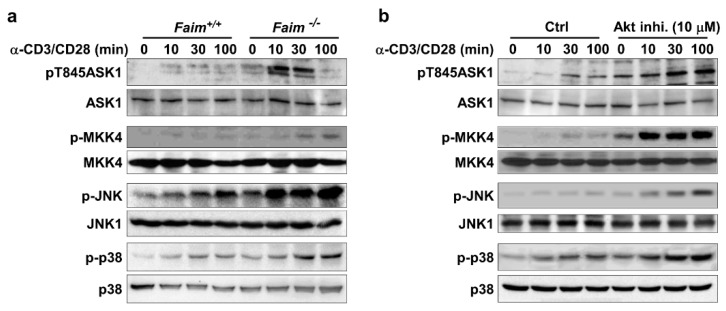
Akt negatively regulates ASK1-JNK/p38-Nur77 signaling in TCR-stimulated thymocytes. (**a**) FAIM-deficient thymocytes exhibit an enhanced activation of ASK1, MKK4, JNK, and p38 upon TCR engagement. Thymocytes from *Faim^+/+^* and *Faim^−/−^* mice were stimulated with anti-CD3 (10 μg/mL) and anti-CD28 (1 μg/mL) antibodies for different periods of time, as indicated. Whole cell lysates were prepared and subjected to immunoblotting for the detection of the phosphorylation status of ASK1, MKK4, JNK, and p38. (**b**) The inhibition of Akt leads to enhanced ASK1-MKK4-JNK/p38 signaling in TCR-stimulated thymocytes. Thymocytes were stimulated with anti-CD3 (10 μg/mL) and anti-CD28 (1 μg/mL) antibodies for different periods of time as indicated, in the absence or presence of the Akt inhibitor. The phosphorylation status of ASK1, MKK4, JNK, and p38 was examined, as described in Figure 4a. The amount of ASK1, MKK4, JNK1/2, and p38 was also detected and included as loading controls for the individual proteins. The data shown are representative of more than four independent experiments.

Next, we sought to understand how ASK1-MKK4-JNK/p38-Nur77 signaling was regulated upon TCR stimulation of thymocytes. As it was previously demonstrated that Fas inhibitory molecule (FAIM) is important for TCR-induced upregulation of Nur77 in thymocytes [23], we asked if FAIM could play a role in modulating ASK1-JNK/p38-Nur77 signaling axes. First, we examined the activation status of ASK1 in TCR-stimulated thymocytes from wild type and FAIM-deficient mice. It was found that TCR stimulation induced much stronger ASK1 phosphorylation at Thr845 in thymocytes lacking FAIM (Figure 4a).

Consistent with this, the phosphorylation of the downstream MKK4, JNK, and p38 MAPK were also augmented in FAIM-deficient thymocytes upon TCR stimulation (Figure 4a), suggesting FAIM could negatively modulate ASK1-MKK4-JNK/p38 signaling.

Our previous study showed that, in TCR-stimulated thymocytes, FAIM regulates the activation and lipid raft localization of Akt [23]. Akt is a pro-survival signaling molecule and is essential for thymocyte development [34]. Interestingly, Akt was shown to negatively regulate ASK1 activation in cells under oxidative stress [19]. Thus, we asked if the activation of Akt, which is facilitated by FAIM, could be involved in ASK1-MKK4-JNK/p38 signaling and Nur77 upregulation during TCR-induced apoptosis of thymocytes. To this end, we treated thymocytes with Akt inhibitor and examined ASK1 activation upon TCR stimulation. It was found that the activation of ASK1, as indicated by its phosphorylation, was much higher in thymocytes treated with the Akt inhibitor (Figure 4b). Consistent with the hyperactivation of ASK1, the activation of MKK4, JNK and p38 was also enhanced in TCR-stimulated thymocytes when Akt was inhibited. In addition, we previously showed that Nur77 level is elevated in the FAIM-deficient thymocytes which have a defective TCR-induced Akt activation [23]. Taken together, these data suggest that FAIM-mediated Akt activation negatively regulates ASK1-MKK4-JNK/p38-Nur77 pathways in TCR-stimulated thymocytes to promote the survival of thymocytes.

## 4. Discussion

**Figure 5:**
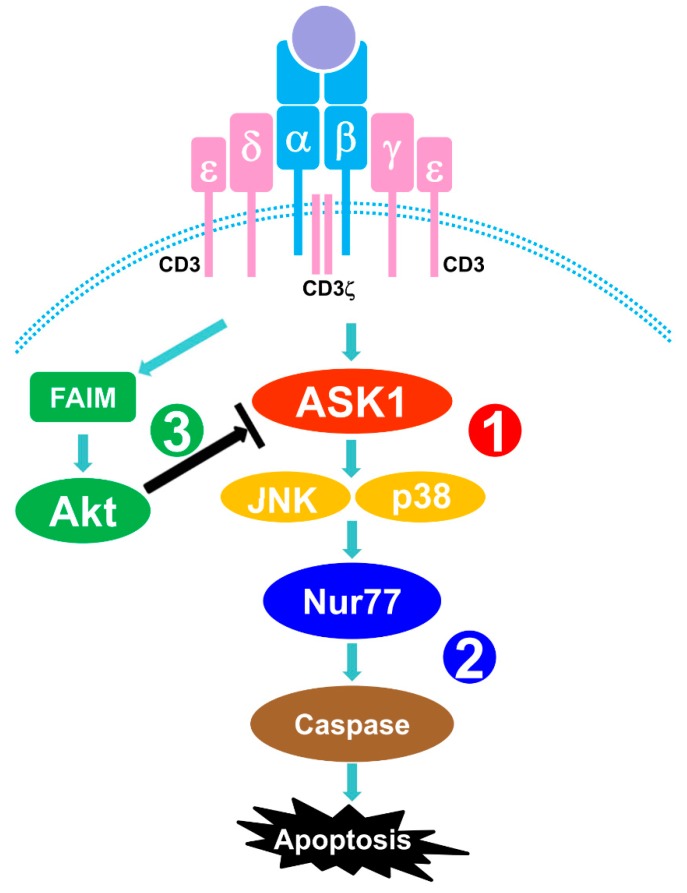
Schematic representation of ASK1-JNK/p38-Nur77 signaling cascades in TCR-stimulated thymocytes. (1) Upon engagement of TCR, ASK1 is phosphorylated and further activates JNK and p38 MAP kinases. (2) Nur77 expression is upregulated by TCR-ASK1-JNK/p38 signaling and is required for caspase activation, leading to the apoptosis of thymocytes. (3) Akt is activated upon TCR stimulation in a FAIM-dependent manner and negatively regulates ASK1-JNK/p38-Nur77 signaling.

In this report, we demonstrate that MAP3K ASK1 is involved in TCR-mediated apoptosis of thymocytes. TCR stimulation triggers the activation of ASK1, which subsequently activates MKK4 and downstream JNK and p38 MAPK signaling cascades that lead to the apoptosis of thymocytes. Mechanistically, TCR-induced activation of ASK1-JNK/p38 signaling axes upregulates the level of Nur77, which is known to play an important role in TCR-mediated apoptosis. Our study further reveals that Akt acts upstream, with the help of FAIM, to dampen the activation of ASK1-JNK/p38 signaling pathways. Thus, our results uncover a new signaling mechanism, whereby ASK1-JNK/p38-Nur77 signaling axes mediate TCR-induced apoptosis of thymocytes and whereby Akt plays an anti-apoptotic role by negatively regulating the activation of ASK1 (Figure 5).

ASK1 is known to be activated by various cytotoxic stresses, including TNF, Fas, reactive oxygen species, UV radiation, osmotic shock, and heat shock [35]. Here, we demonstrated that ASK1 is also activated rapidly in thymocytes upon TCR stimulation. Interestingly, its activation kinetics is similar to that of its downstream targets JNK and p38 MAPK, suggesting that they are TCR proximal signaling events which occur promptly upon TCR engagement. Previous studies demonstrated that the activation of ASK1 is regulated by thioredoxin, calcium, and integrin binding protein 1 in a redox- and calcium-dependent manner [36]. It is known that TCR stimulation triggers calcium flux and oxidative phosphorylation, leading to the proliferation and activation of T cells [37]. Hence, it is possible that strong stimulation of TCR with high affinities for self-antigens triggers the calcium signaling and redox reaction resulting in ASK1 activation and that the cells undergo cell death instead of activation and proliferation.

We further show that Nur77 is the target downstream of ASK1-JNK/p38 pathways during TCR-induced apoptosis of thymocytes. It is known that the expression of Nur77 is induced in response to the strong engagement of TCR and that it is involved in thymocyte apoptosis and negative selection [38]. Interestingly, Nur77 has been demonstrated to play a proapoptotic role in a mitochondrial-dependent manner [29]. This is consistent with the findings that the overexpression of wild-type or constitutively active ASK1 also induces apoptosis through mitochondrial-dependent caspase activation in cells under various cytotoxic stresses [28,39,40]. The level of Nur77 in TCR-stimulated thymocytes is dependent on the level of ASK1 or the activation status of ASK1, JNK, or p38 (Figure 3), suggesting that the signals propagated through ASK1-JNK/p38 cascades converge at Nur77 and are further translated into an apoptotic signal. It is very likely that the same signaling ASK1-JNK/p38-Nur77 axes also exist in cells in response to stress signals, in such a way that Nur77 upregulation or stabilization account for the beginning of cell death. In addition, the more complete suppression of Nur77 by ASK1 inhibition, compared to that by individual or combinatorial inhibition of JNK and p38, suggests that other pathways downstream of ASK1 could also contribute to Nur77 regulation. In line with this observation, a previous study showed that the MEK5-ERK5 signaling pathway also targets Nur77 during TCR-induced apoptosis of T cells [33].

Our previous study showed that FAIM-deficient thymocytes had an elevated level of Nur77 due to defective Akt activation upon TCR stimulation [41]. Here, we further show that both the FAIM-deficient thymocytes, which have a defective Akt activation, and wild-type thymocytes with Akt inhibited, have an enhanced activation of ASK1-JNK/p38 signaling cascades. These results suggest that Akt negatively regulates ASK1-JNK/p38 signaling in TCR-stimulated thymocytes. This is reminiscent of the finding that Akt negatively regulates ASK1 activation in cells undergoing oxidative stress [19]. Taken together, our studies suggest that the negative regulation of ASK1 by Akt could be a general phenomenon in apoptotic cells. In summary, our study demonstrates that ASK1-JNK/p38-Nur77 are important signaling axes for the TCR-induced apoptosis of thymocytes and that Akt negatively modulates these pathways.

## Supplementary Materials

The following are available online at https://www.mdpi.com/2073-4409/9/3/585/s1, Figure S1. Relative optical density (OD) from immunoblots of Figure 1b,c. The blots of ASK1 and cleaved caspase-3 (C.Casp3) were semi-quantitatively analyzed by Image J software, and the sum optical density was obtained. Values are ratios to signals that were obtained for loading control β-actin. The data are the means ± SEM of three experiments. Figure S2. Relative optical density (OD) from immunoblots of Figure 2c. The blots of JNK and p38 phosphorylation were semi-quantitatively analyzed by Image J software, and the sum optical density was shown as a bar graph. Values are ratios to signals that were obtained for the loading controls JNK and p38. The data are the means ± SEM of three experiments. Figure S3. Relative optical density (OD) from immunoblots of Figure 3c. The blots of Nur77 were semi-quantitatively analyzed by Image J software, and the sum optical density was shown as a bar graph. Values are ratios to signals that were obtained for loading control β-actin. The data are the means ± SEM of three experiments.

## Abbreviations

AKT: AKR mouse thymoma kinase; ASK1 or MAPKKK5, apoptosis signal-regulating kinase 1;Bad, Bcl-2-associated death promoter; C. Casp3, cleaved caspase-3; ERK, extracellular signal–regulated kinase; DLK, dual leucine zipper-bearing kinase; DN, double negative; FAIM, Fas Apoptosis Inhibitory Molecule; GSK-3, glycogen synthase kinase 3; IL-1, interleukin-1; JNK, the c-Jun NH2-terminal kinase; PI, propidium iodide; MAP3k, MAPKK kinase; MAPK, Mitogen-activated protein kinases; MAPKK, MAPK kinase; MKK6, MAPK kinase 6; MLK2, mixed-lineage kinase-2 (MLK2); MLK3, Mixed lineage kinases; MST, mammalian STE20-like kinases; MTK1, MAP Three Kinase 1; MKK4, mitogen-activated protein kinase kinase 4; MUK, mitogen-activated protein kinase upstream protein kinase; NF-κB, nuclear factor-kappaB; Nur77, neuron-derived clone 77; OD, optical density; PI, propidium iodide; PTK, protein tyrosine kinase; PVDF, polyvinylidene difluoride; self-pMHC, SEK1, SAPK/ERK kinase 1; self-pMHC, self peptide and major histocompatibility complex proteins; siRNA, small interfering Ribonucleic Acid; SPRK, src-homology 3 (SH3) domain-containing proline-rich kinase; TAK1, TGF-beta-activated kinase 1; TCR, T cell receptor; ZPK, leucine-zipper protein kinase.
