# Supplementary material for: ASK1 Mediates Nur77 Expression in T-Cell Receptor Mediated Thymocyte Apoptosis
Source: Cells. 2020 Mar 1;9(3):585. doi: 10.3390/cells9030585 (PMC7140521; doi:10.3390/cells9030585)
Supplement: Supplementary file 1 [file cells-09-00585-s001.pdf]

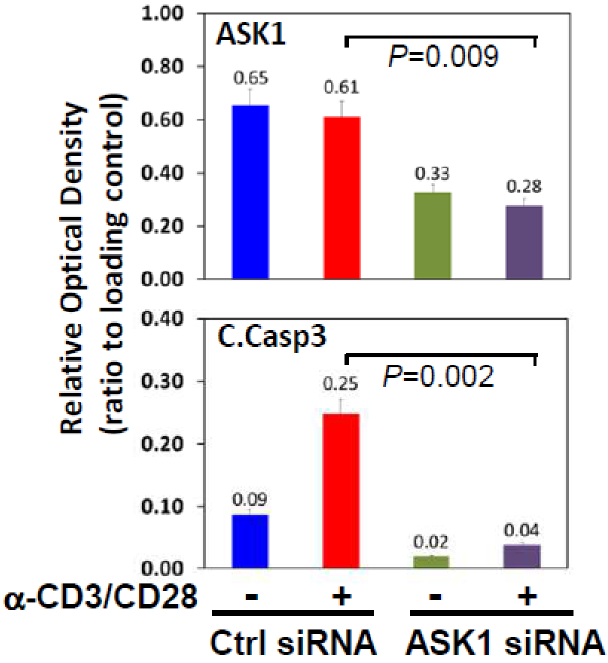

Supplementary Figure S1. Relative optical density (OD) from immunoblots of Figure 1b and 1c. The blots of ASK1 and cleaved caspase-3 (C.Casp3) were semi-quantitatively analyzed by Image J software and the sum optical density was obtained. Values are ratios to signals that were obtained for loading control ERK2. Data are means ± SEM of 3 experiments.

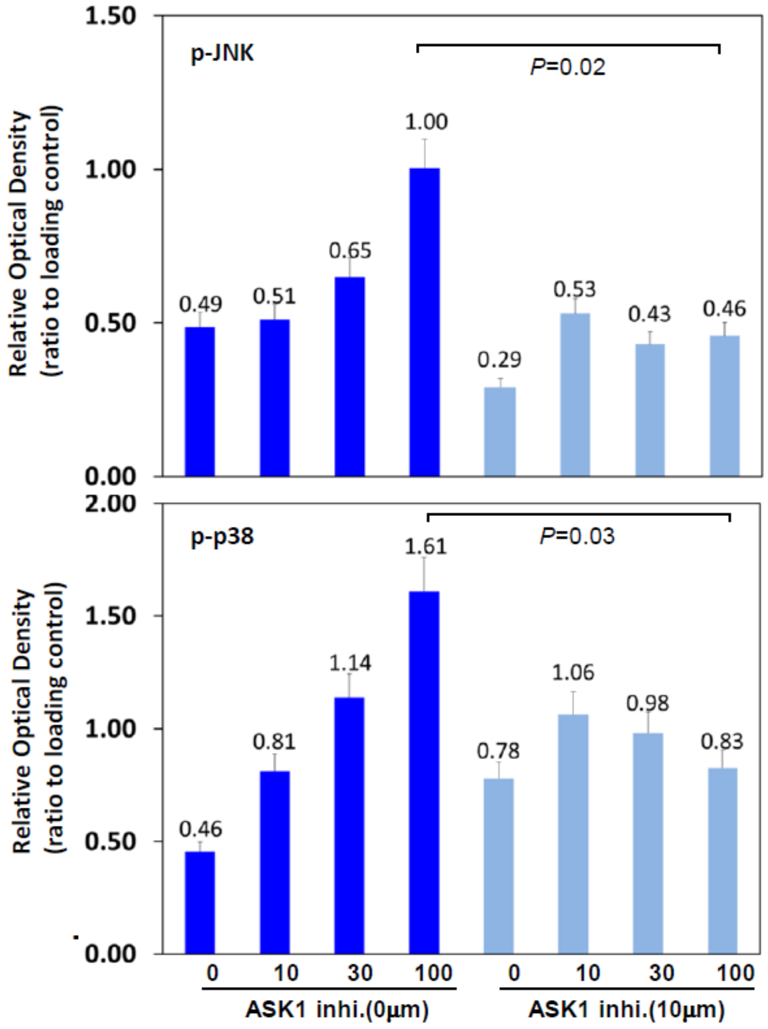

Supplementary Figure S2. Relative optical density (OD) from immunoblots of Figure 2c. The blots of JNK and p38 phosphorylation were semi-quantitatively analyzed by Image J software and the sum optical density was shown as bar graph. Values are ratios to signals that were obtained for loading control JNK and p38. Data are means ± SEM of 3 experiments.

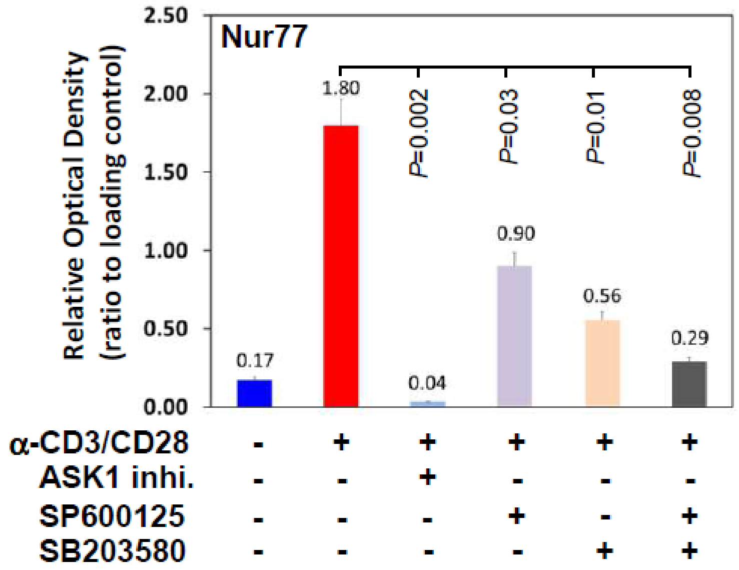

Supplementary Figure S3. Relative optical density (OD) from immunoblots of Figure 3c. The blots of Nur77 were semi-quantitatively analyzed by Image J software and the sum optical density was shown as bar graph. Values are ratios to signals that were obtained for loading control ERK2. Data are means ± SEM of 3 experiments.
